# Student evaluation of a primary care clerkship: quality assurance and identification of potential for improvement

**DOI:** 10.1186/1472-6920-9-17

**Published:** 2009-04-15

**Authors:** Jean-François Chenot, Michael M Kochen, Wolfgang Himmel

**Affiliations:** 1 Department of General Practice/Family Medicine, University of Göttingen, Humboldtallee 38, 37073 Göttingen, Germany

## Abstract

**Background:**

In Germany, like many other countries, general practice clerkships have only recently become mandatory during medical education. The biggest challenges for the organisation of such clerkships are achieving a minimum level of standardisation, and developing and maintaining a system of quality assurance. The aim of this study is to assess the instructional quality in teaching practices using a benchmark system.

**Methods:**

Before commencing, students anonymously assessed the importance of core aspects of the mandatory primary care clerkship. After the clerkship, they evaluated learning opportunities and teaching performance. Based on this data, a benchmark system was developed to identify areas of strength and weakness for all practices as well as individual teaching practices.

**Results:**

A total of 695 students evaluated 97 general practices belonging to a teaching network. Prior to the clerkship, most students considered recognition of frequent diseases (85%) and communication skills (65%) the most important learning goals. After the clerkship, nearly 90% of students confirmed that the general practitioner (GP) was good or excellent at teaching these two goals but only two-thirds thought the GP's teaching performance good or excellent in preventive medicine and screening. In an exemplary analysis, we identified the 2 best and the 2 worst practices that consistently received scores far above or below average, respectively.

**Conclusion:**

We were able to identify areas of weakness in teaching and identified specific GPs who did not meet the students' needs and expectations. This evaluation seems to be a useful quality assurance tool to identify the potential for improvement and faculty development.

## Background

There has always been a gap between the number of patients seen in primary care and how this is reflected in the curriculum of medical schools. To overcome this shortcoming, mandatory primary care clerkships are, to date, a component of most curricula. In the United States and the United Kingdom, for example, many schools already deliver a significant proportion of their curricula in the community [1-3]. However, in Germany – like many other countries – general practice is still not fully established in most medical schools, and clerkships in primary care/general practice were not mandatory until recently. In early 2004, the 8^th ^amendment of the Federal Regulations for Medical Education in Germany instituted mandatory practical training in primary care [4]. Clerkships in primary care in German medical schools range from 1 to 3 weeks. A 2-week clerkship in primary care in the 5^th ^year was introduced in 1999 at the University of Göttingen Medical School.

The core curriculum for this clerkship focuses both on general principles of teaching and on specific tasks of general practice. Some of the essentials are:

▪ Communication skills, recognising patient expectations

▪ Upper respiratory tract infections

▪ Home visits and emergencies

▪ Chronic disease, multi-morbidity and palliative care

▪ Sick leave

▪ Functional and psychosomatic complaints

▪ Musculoskeletal disorders, e.g. low back pain

▪ Preventive care in primary care

▪ Appropriate prescribing

Some of these tasks also reflect learning objectives, e.g. recognising and treating upper respiratory tract infection or communications skills, which fall within the curricular responsibility of family medicine in our faculty.

Primary care practices are usually rather loosely attached to medical schools. The instructional quality of undergraduate medical education in the community setting has been described as a "black box" [5] where the quality of teaching in some practices and the availability of time to teach and offer patient care are matters of concern [1]. This is due to variation in practice structure as well as the large number of decentralised sites (compared to a hospital setting) and the high number of tutors. The biggest challenges are creating and maintaining a minimum level of standardisation and a system of quality assurance [6,7]. Clerkship students are expected to learn from tutors, not usually trained in teaching, core concepts of primary care, such as "whole person" and "humanised health care" [8], along with strategies for differential diagnosis, patient management and comprehensive care for chronic illness [9]. Training programmes for general practitioners (GPs) with a special interest in medical education have been established elsewhere [10] but are either not available in Germany or restricted to doctors who are highly motivated to take part in teach-the-teacher training courses [11,12].

Although teaching general practices seem to perform better, for example, in prevention activities or prescribing indicators than non-teaching practices [13], the quality of community-based education cannot be taken for granted [6]. As early as 1999, Shipengrover and James [5] complained about a lack of valid measures to monitor the quality of teaching during clerkships. Meanwhile, there are some measures to survey the students' most valued practice and preceptor characteristics and to evaluate instructional quality in ambulatory teaching sites [6,14,15]. However, these measures lack a focus on characteristics unique to primary care.

A student assessment may provide exactly such data continuously, namely process data, which is needed to evaluate the quality of medical education. We therefore introduced a mandatory evaluation with a special focus on primary care characteristics that could form the basis of a subsequent benchmarking system. The aims of this study were to explore:

(1) Which expectations or preconceptions students had of a primary care clerkship.

(2) Which areas of teaching students did and did not appreciate to identify GPs' areas of strength and weakness in teaching.

(3) Correlations between the students' evaluation of the clerkship and their desire to pursue a career in primary care.

(4) Whether the data helped to identify practices with excellent or only moderate teaching performance.

## Methods

### Teaching physicians/preceptors

Most primary care in Germany is delivered by GPs in private practices. The recruitment of teaching GPs started in 1999 from all GPs and some internal medicine primary care providers in the district surrounding the Göttingen University Medical School. All general practitioners in Germany have a board certified specialty training, which requires 3 to 5 years postgraduate training. They can apply for their practice to become a teaching site and become a member of the general practice teaching network if they agree to comply with organisational requirements as well as the learning content. These pre-requisites are displayed on the department's homepage [16]. The most important requirements are at least 3 years in practice, a second consultation room allowing students to see patients by themselves, and a focus in regular primary care. Practices providing for example mainly naturopathic medicine are excluded.

The GPs are kept informed of current requirements by e-mail and an annual pamphlet. So far, we do not require teaching GPs to attend a mandatory course in medical education. GPs continue to see patients while teaching and they receive 25 € per day of student supervision to cover for the extra time students require to discuss patient care and examine patients Additionally the practice receives the title "academic teaching practice".

Compared with the demographic data of the doctors in the district of Göttingen, Lower Saxony, the average age of our teaching GPs did not differ significantly (51.3 vs. 52.3 years); the proportion of women was slightly smaller (24.1% vs. 20.4%). Nearly 60% of all teaching GPs were working single-handed and about 40% in group practices [17].

### Medical students

In Germany, medical school training requires 6 years (2 years of basic science and four years of clinical coursework). Students are required to select a practice from the teaching network for a 2-week clerkship. It is recommended that the clerkship be taken at the beginning of the 5^th ^year. However, students are allowed to do it earlier if, for example, they plan a year abroad. A tutorial with case-based learning using the experience of students gained during the clerkship takes place at the end of the 5^th ^year.

### Data collection

In April 2003, a mandatory web-based questionnaire was introduced both prior to (pre-evaluation) and after the clerkship (post-evaluation). This is a self-developed questionnaire based on the learning objectives of the core curriculum. The evaluation questionnaires, including a test version, can be accessed via the departmental website [18]. Students identify themselves with their student identification number over a secure connection. This number is used to match the pre- and post-questionnaires. Only completed questionnaires can be returned via the Internet. Personal data is stored separately from the questionnaire. The technical details have been described elsewhere [19].

The questionnaires allow the students to rate the importance of key aspects of the core curriculum of the primary care clerkship (pre-evaluation) as well as an assessment of the teaching practice and the tutor's performance (post-evaluation). Relevant items can be seen in Table 1. Students are also asked after their clerkship if they feel encouraged to pursue a career in primary care.

**Table 1:** Student assessment of the importance of key aspects of the core curriculum before the clerkship and the post-clerkship evaluation*

	Importance; rated before clerkship			Evaluation, after clerkship			
		
	Very important	Important	Less important	Excellent	Good	Sufficient	Insufficient
Key aspect	%	%	%	%	%	%	%
	
recognition of frequent diseases	85	15	-	40	47	10	3
prescriptions of medicine	43	50	7	22	51	20	7
preventive medicine	46	46	8	24	41	23	12
screening	48	45	7	28	36	23	13
vaccinations	25	52	23	31	37	21	11
family medicine	25	54	21	32	40	17	11
home visits	29	49	22	45	35	13	7
communication skills	65	30	5	50	37	10	3
patient expectations	31	59	10	35	49	13	3
caring for chronic patients	41	51	8	33	46	16	5
physical examination	61	30	9	29	44	18	9
collaboration with specialists	26	54	20	17	40	28	15

*based on n = 695 students

### Data analysis

We analysed all questionnaires that were returned via the Internet during a 2-year period from April 2003 to March 2005. Most items on both evaluation forms were provided with a rating scale as an answer format. The results of the evaluation are reported as relative frequencies in the case of 3- and 4-point-scales, and – for reasons of convenience – as mean values (with their standard deviation [SD]) in the case of 10-point scales.

To rank practices according to the students' evaluations, we assessed the proportion of items rated as excellent or good per practice. We only ranked practices that had been evaluated by at least 5 students. To determine the internal coherence of the post-evaluation form, we calculated Cronbach's alpha for each practice. The Wilcoxon rank test was used to compare the students' overall rating of the clerkship quality depending on whether they felt encouraged to pursue a career in primary care or not. The software package SAS 9.1 was used for analysis.

## Results

From a network of 125 teaching practices, 97 GPs had supervised at least one student during the study period (mean per practice: 7.2; median: 5; range: 1 to 35). Fifty-one practices had at least 5 students. At the time of the data analysis, a total of 695 students (44% female) had evaluated the primary care clerkship via the Internet. We had no missing data since a complete evaluation is a prerequisite for receipt of the clerkship certificate.

Table 1 shows how the students assessed the importance of various aspects of the core curriculum *before *the clerkship and how well these same aspects had been addressed *after *the clerkship. "Recognition of frequent diseases" was considered the most important characteristic. "Physical examination" and "communication skills" were considered important by many students before the clerkship and received good scores in the evaluation after the clerkship. "Vaccinations", "family medicine", "collaboration with specialists" were considered to be less important and received a less positive evaluation, whereas "home visits", also not considered to be important by many students, was rated excellent.

Table 2 shows how the students judged the tutors' didactic performance. Most students were (highly) satisfied with the total quality of the clerkship (mean score: 8.1 on a 10-point-scale). They felt the doctors spent enough time (mean score: 8.3) and the demands were adequate (8.9). However, in the opinion of the students, the strategies adopted for "problem solving" in primary care were less well taught (7.1).

**Table 2:** Student ratings of the tutors' didactic performance

Teaching performance	Mean (SD) *	Range	clerkships rated below 7* (%)
The preceptor took time to discuss tasks assigned to me.	8.3 (2.2)	1–10	17%
I had enough time to acquire new skills.	8.0 (2.4)	1–10	19%
The clerkship was not too theoretical.	7.6 (1.9)	1–10	17%
The demands were adequate.	8.9 (1.8)	1–10	12%
I learned strategies for problem solving.	7.1 (2.3)	1–10	30%
Overall I am satisfied with the quality of the clerkship.	8.1 (2.5)	1–10	17%

*on a 10-point -scale (1 = disagree; 10 = strongly agree)

**Figure 1:**
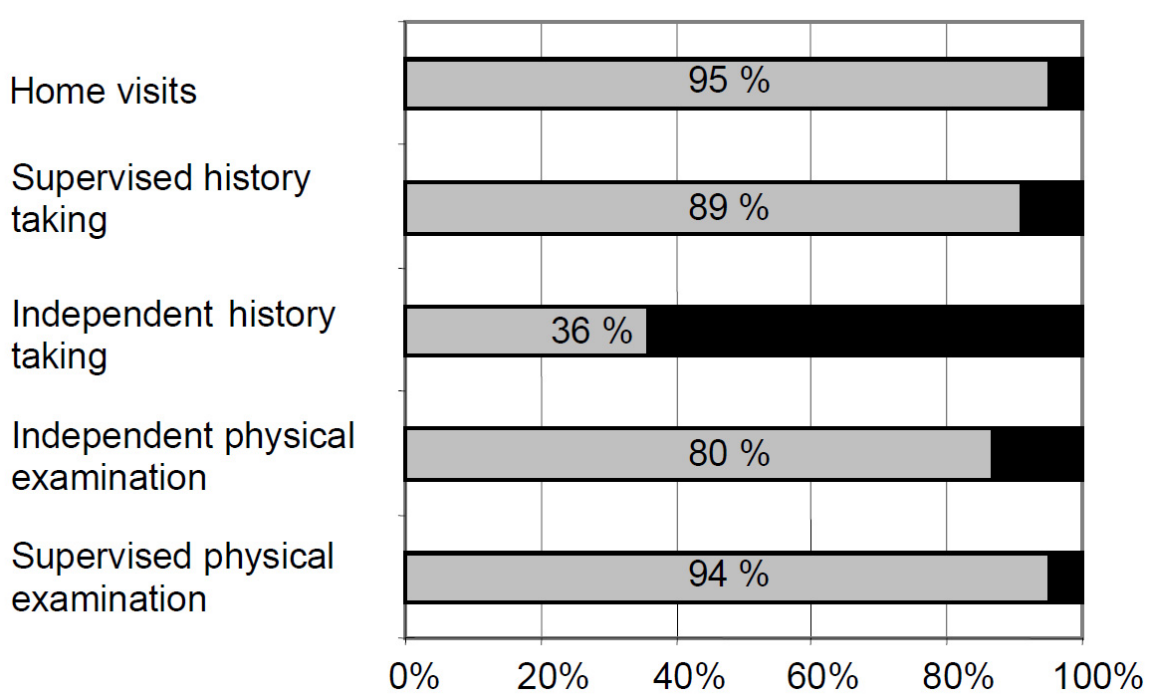
**Learning opportunities for students**. Yes: Grey shading. No: Black shading.

Figure 1 shows the opportunities for students to learn and gain practical experience during the clerkship. Most of them had the opportunity to perform one or several independent physical examinations during the clerkship, but in more than half of the clerkships, the students were either not allowed or did not feel encouraged to take a history without the presence of their tutor.

Some GPs received excellent scores in all or most of the performance indicators. In a sample analysis, we identified the two best and the two worst practices (Table 3). Seventeen students did their clerkship in the two top practices. Of theses students, on average 94%, respectively 93% rated all teaching items as either good or excellent. For 8, respectively 7 items, all students in one practice agreed that the teaching was good or excellent. In contrast, on average only 31%, respectively 54% of the students who did their clerkships in the two worst practices found the teaching to be either good or excellent. Two items of practice 43 – one of the worst practices – were rated as good or excellent by 70% and 80% of the students. This percentage slightly exceeded the average rate for all practices. However, most items were rated far below average for both of these "bottom practices" and no items of the core curriculum were unanimously found to be good or excellent. Evaluation of the different students in one practice was consistent for most performance characteristics, with correlations (Cronbach's alpha) for all 51 practices, which were evaluated by at least 5 students, ranging from 0.75 to 0.94, with an average of 0.91.

**Table 3:** Contrasting the proportion of students who rated the teaching of the items from the core curriculum as excellent or good (highest versus lowest ranked practices).

	Top Practices		Bottom Practices		All Practices
			
	Practice 44	Practice 82	Practice 43	Practice 108	
	9*	8*	20*	10*	695
Characteristic	(%)	(%)	(%)	(%)	(%)
recognition of frequent diseases	100**	**100**	45	50	88
prescriptions of medicine	**100**	**100**	30	40	72
preventive medicine	**89**	**87**	40	20	65
screening	**100**	**75**	**70**	40	68
vaccinations	**100**	**100**	65	40	71
family medicine	**78**	**100**	30	20	65
home visits	**100**	**87**	**80**	30	79
communication skills	**100**	**100**	75	40	87
patient expectations	**88**	**100**	80	50	84
caring for chronic patients	**88**	**87**	50	10	78
Physical examination	**100**	**100**	65	20	73
collaboration with specialists	**88**	**100**	20	10	58
average	**94**	**93**	54	31	74
Cronbach's alpha	0.86	0.83	0.92	0.94	0.91

* Number of students who evaluated the practice

** Bold: above average evaluation

Students who had a good experience during the clerkship and rated practices favourably were significantly more likely to consider pursuing training in primary care. These students (n = 374) rated the quality of the clerkship with an average score of 8.8 (CI_95 _8.6–9) compared to an average score of 7.2 (CI_95 _6.9–7.5) given by the remainder (n = 321).

## Discussion

### Findings

Most students considered recognition of frequent diseases, communication skills and physical examination very important learning goals before starting their primary care clerkship. According to the students' assessment, teaching GPs did a good job conveying these central goals, but were less successful, for example, in conveying the importance of preventive medicine, screening or collaboration with specialists. With the exception of independent history taking, most students reported that they had had sufficient learning opportunities. On average, students were satisfied with the teaching performance, except when it came to problem solving strategies. However, we were able use this benchmarking system to identify teaching physicians who consistently received ratings far below average.

### Strengths and limitations of the study

Our results are based on large samples of students and teaching GPs and may therefore be applicable to all academic teaching practices in Germany. We believe that the establishment of a benchmark system to evaluate teaching performance in ambulatory care is of interest outside Germany.

All student evaluations are based on the hypothesis that students are the best experts to assess their teachers [20]. Nevertheless; we did not study the adequacy of the assessments made by our medical undergraduates. We also appreciate that while students might be reluctant to award unfavourable ratings after two weeks of close contact, about 10% of the GP tutors did receive poor evaluations for their teaching performance. However, to be protected against highly subjective and undue opinions, we considered in our benchmark scenario only practice ratings from at least five students. Standardised medical student evaluations could represent an alternative to assess the quality of GP teaching [21] but this approach would have required more substantial funding.

It is unclear how many students are needed to obtain a valid assessment of a teaching physician; we arbitrarily used a cut off of five students [22]. This number might be too low since seasonal factors, the availability of patients and interpersonal factors could possibly influence the quality of teaching and thus the students' evaluations. Even so, the scores given by students who did their clerkship in the same practice did not vary greatly, so that the identification of poor practices does not seem to be the result of an arbitrary or random assessment.

The strong association between practice assessment and anticipated careers in primary care can, of course, not be interpreted as a strict causal path. Some of the students considering primary care as a career might have been positively biased towards the primary care clerkship whereas others interested in a different specialty might have felt bored by any experience in primary care, independent of their tutor's teaching performance. In a Slovenian long-term evaluation, high scores in student satisfaction with family medicine did not influence the career choice [23]. The association between practice assessment and career aspirations found in our study is in line with a recent systematic review of the positive outcomes of early exposure to clinical and community settings on career choices in primary care specialties [24].

### Context

According to a US study [6] and a Canadian study [25], the following attributes are essential for educational quality in ambulatory teaching and valued by students: open communication with the teacher, feedback, integration of the student into the clinical setting, explaining clinical reasoning, improvement of communication skills and giving students an active role in patient care. Most of these attributes were also highly valued by our students, especially communication skills (considered important or very important by 95% of our sample).

Compared to the other measures of the students' most valued practice and teacher characteristics [14,26], our questionnaire also addressed core aspects of primary care. More than 20% of the medical undergraduates in our sample did not consider the following key elements of primary care and family medicine [27] to be important: vaccinations, family medicine, home visits and collaboration with specialists. Additionally, nearly two-thirds of the students did not have any chance to take "patient history independently" during the clerkship. It is exactly this opportunity that has been found to be an important factor for students' enthusiasm in the above-mentioned Canadian study [25].

Summarising the literature, Howe [2] names several important consequences of learning in a primary care setting, e.g. retention of patient-oriented values more effectively than in previous curricula and enabling early application of theory into clinical practice. However, without continuous evaluations, we cannot be sure as to whether these beneficial effects really occur in all primary care clerkships. Our methodology may therefore represent a fair and valid benchmarking system that identifies poor teaching practices.

### Implications for practice and research

We need to improve teaching in some core items, namely preventive medicine, screening and problem solving strategies and to increase opportunities for independent history taking during the primary care clerkship. Qualitative studies may also help us to better understand why many students rate "family medicine" rather low. Once these reasons are uncovered, we will then be able to better encourage students to consider a career in primary care.

Since feedback and evaluation from students are usually highly valued by community-based preceptors [28], teaching GPs should receive a standardised feedback comparing their performance with the overall performance of all practices on each evaluated topic. According to the experience of a student feedback study from Turkey [29], students' ratings should be reported more than once a year to have impact on the instructors' teaching performance. For identified areas of weakness, the distribution of a brief faculty development audiotape, as suggested by Willett [30] may be helpful, especially for a dispersed group of ambulatory preceptors who find it difficult to attend seminars. Practices that consistently receive excellent evaluations should receive public recognition.

To our knowledge, there is no agreed standard defining what proportion of dissatisfied students is acceptable. If more than 30% of students rate an item of the core curriculum as sufficient or poor, we assume that this is a call for action. We also believe that students' assessments (with coefficients of reliability between 0.75 and 0.94) are consistent enough to rank practices according to their teaching performance. Both assumptions should be validated by further research.

An important and challenging issue for further studies is how to address those practices that perform below average. To our knowledge, nothing has been published on this delicate issue. Current attempts focus typically on programmatic evaluation rather than improving quality through validated benchmarks. To study the consequences, if any, of practice benchmarking combined with a feedback process, could help continuously monitor and improve faculty development [31].

## Conclusion

Benchmarking teaching practices based on student evaluations may help to identify areas of weakness in teaching and deficient teaching practices. These results could be used as a basis for tailored educational interventions and may improve the quality of primary care clerkships.

## Abbreviations

(GP): General practitioner.

## Competing interests

The authors declare that they have no competing interests.

## Authors' contributions

JFC, MMK, WH conceived and outlined the online evaluation procedure. JFC and WH developed the online benchmarking system and designed the study, collected the data and wrote the first draft. All authors participated in the writing and editing process and approved the final manuscript.

## Pre-publication history

The pre-publication history for this paper can be accessed here:


